# Identification of two new recessive *MC1R* alleles in red‐coloured Evolèner cattle and other breeds

**DOI:** 10.1111/age.13206

**Published:** 2022-04-22

**Authors:** Miriam Hauser, Heidi Signer‐Hasler, Luzia Küttel, Aurélien Capitan, Bernt Guldbrandtsen, Dirk Hinrichs, Christine Flury, Franz R. Seefried, Cord Drögemüller

**Affiliations:** ^1^ Institute of Genetics, Vetsuisse Faculty University of Bern Bern Switzerland; ^2^ School of Agricultural, Forest and Food Sciences (HAFL) Bern University of Applied Sciences Zollikofen Switzerland; ^3^ ALLICE Paris France; ^4^ INRAE AgroParisTech GABI Université Paris‐Saclay Jouy‐en‐Josas France; ^5^ 4321 Department of Veterinary and Animal Sciences University of Copenhagen Frederiksberg C Denmark; ^6^ 9178 Department of Animal Breeding Faculty of Organic Agricultural Sciences University of Kassel Witzenhausen Germany; ^7^ Qualitas AG Zug Switzerland

**Keywords:** *Bos taurus*, coat colour, *ev1*, *ev2*, heterogeneity, melanism, melanocortin‐1 receptor, pigmentation

## Abstract

Sequence variations in the *melanocortin‐1 receptor* (*MC1R*) gene are associated with melanism in different animal species. Six functionally relevant alleles have been described in cattle to date. In a hypothesis‐free approach we performed a genome‐wide allelic association study with black, red and wild‐coloured cattle of three Alpine cattle breeds (Eringer, Evolèner and Valdostana), revealing a single significant association signal close to the *MC1R* gene. We searched for candidate causative variants by sequencing the entire coding sequence and identified two novel protein‐changing variants. We propose designating the mutant alleles at *MC1R*:c.424C>T as *e^v1^* and at *MC1R*:c.263G>A as *e^v2^*. Both affect conserved amino acid residues in functionally important transmembrane domains (p.Arg142Cys and p.Ser88Asn). Both alleles segregate predominantly in the Swiss Evolèner breed. They occur in other European cattle breeds such as Abondance and Rotes Höhenvieh as well. We observed almost perfect association between the *MC1R* genotypes and the coat colour phenotype in a cohort of 513 black, red and wild‐coloured cattle. Animals carrying two copies of *MC1R* loss‐of‐function alleles or that were compound heterozygous for *e*, *e^v1^*, or *e^v2^* have a red to dark red (chestnut‐like red) coat colour. These findings expand the spectrum of causal *MC1R* variants causing recessive red in cattle.

## INTRODUCTION

**FIGURE 1:**
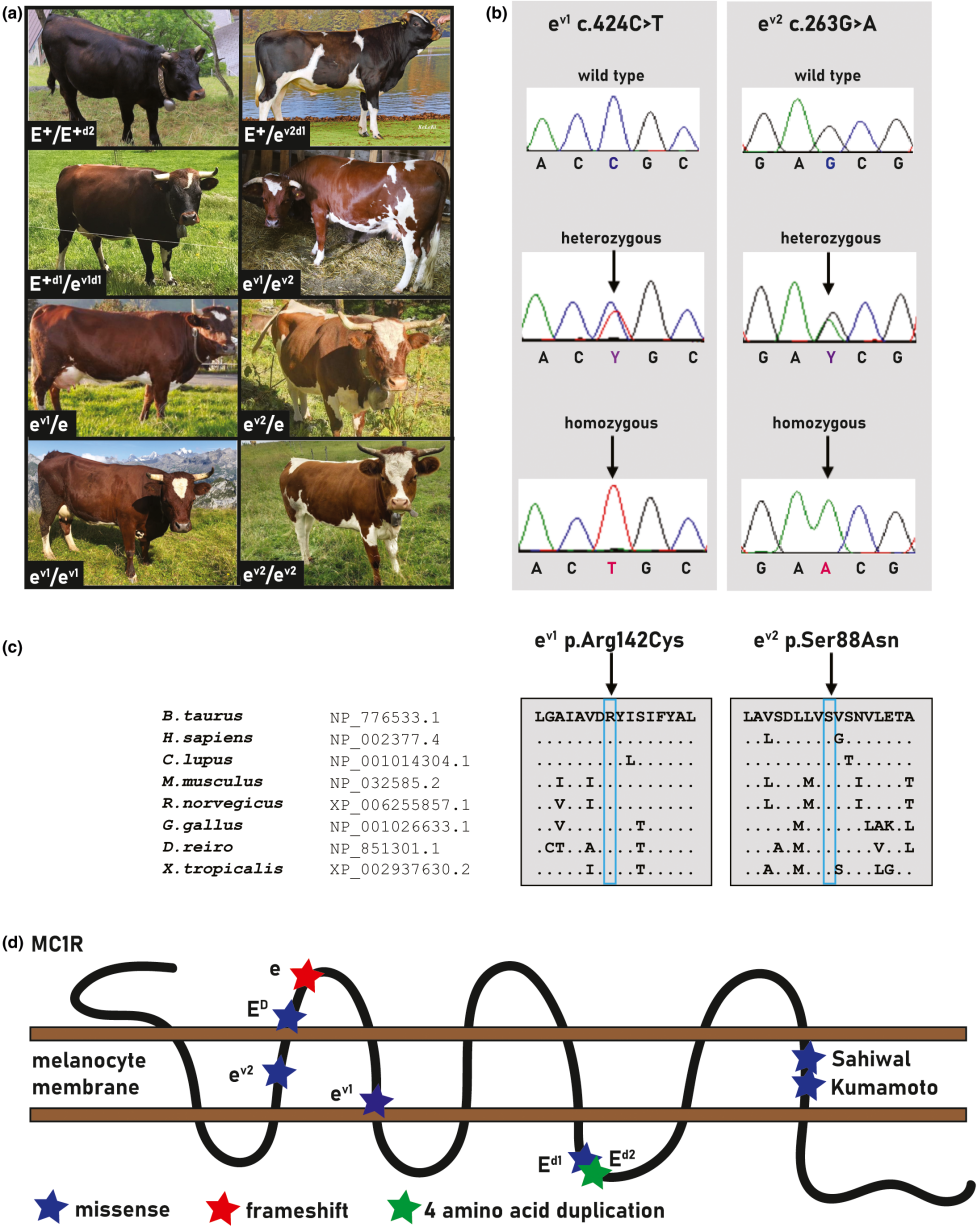
Characteristics of two new functional *MC1R* alleles. (a) Red coat colour in different *MC1R* genotypes. Note the different shades of red in the presence of the *e^v1^* and *e^v2^* alleles compared with wild‐coloured animals homozygous or heterozygous for the *E* ^+^ allele. Note that the muzzles of animals carrying the *e^v1^* allele appear to be darker pigmented. (b) Electropherograms showing the different *e^v1^* and *e^v2^* genotypes obtained by Sanger sequencing. (c) Multiple sequence alignments of the MC1R protein encompassing the region of the p.Arg142Cys and p.Ser88Asn variants demonstrate complete evolutionary conservation across species. (d) Schematic representation of the bovine MC1R protein and its seven transmembrane domains obtained from the UniProt database (http://www.uniprot.org/; accession number: P47798). All known and newly discovered *E* alleles mentioned in this work are shown with asterisks. Note that both missense variants reported in red‐coloured Sahiwal (p.Thr281Asn) and Kumamoto (p.Ala291Thr) cattle also affect conserved residues of a transmembrane domain as in *e^v1^* and *e^v2^*

The individual base coat colour of animals is determined by pigment synthesis through a process called pigment‐type switching within melanocytes (Barsh et al., 2000). In all mammals, the agouti‐signalling protein (ASIP) and its receptor, the melanocortin‐1‐receptor (MC1R), play fundamental roles in this process by modulating the relative synthesis of yellow‐reddish pheomelanin and dark brown or black eumelanin by regulating the level of tyrosinase expression (Ito & Wakamatsu, 2011). Activation of MC1R, a G‐protein coupled receptor with seven transmembrane domains, by its ligand, *α*‐melanocyte stimulating hormone (MSH), induces the melanocytes to produce eumelanin, whereas inhibition of MC1R that occurs when the ASIP protein is expressed leads to the production of pheomelanin (Barsh et al., 2000; Robbins et al., 1993). Hence, genetic variants that alter either *ASIP* or *MC1R* activity can affect the synthesis of pheomelanin or eumelanin (Brancalion et al., 2022). Before these genes were discovered, they were called the *A* (*Agouti*) and *E* (*Extension*) loci. Almost 90 years ago, evidence was reported supporting the existence of a series of multiple alleles at the *E* locus, including the dominant black of Angus and Holstein cattle (Ibsen, 1933). For over 25 years it has been known that solid yellow or red cattle are homozygous for a recessive loss‐of‐function allele in *MC1R*, denoted *e*, caused by the p.Gly104ValfsTer53 variant. This allele results in the sole production of pheomelanin pigment, whereas a dominant gain‐of‐function allele in *MC1R*, denoted *E^D^*, caused by the amino acid exchange p.Leu99Pro, is responsible for eumelanin expression of solid black cattle (OMIA 001199‐9913; Jörg et al., 1996; Klungland et al., 1995; Kriegesmann et al., 2001). In addition to the two globally occurring derived alleles *e* and *E^D^*, the ancestral *MC1R* allele designated as *E* ^+^ has been reported in various breeds of cattle (Olson, 1999). The order of dominance is *E^D^* > *E* ^+^ > *e* (Lawlor et al., 2014; Olson, 1999). Cattle that are *e*/*e* are red and *E^D^*/− are typically black, as *E^D^* is the dominant allele and eumelanin is permanently produced. Individuals with the genotypes *E* ^+^/*E* ^+^ and *E* ^+^/*e* can be of any colour as *E* ^+^ acts as a neutral allele and normal activity of the melanocortin‐1 receptor is assumed so that both types of pigment are produced simultaneously in different parts of the body (Figure 1a; Figure S1c; Klungland et al., 1995). It is speculated that the *E* ^+^ allele predominantly occurs in the ancestral breeds of the present‐day cattle have more pheomelanin content and that, over time, mutations have introduced more variations, leading to many shades. This could have occurred either because of interactions or because of deletions in the responsible genes (Seo et al., 2007). In the meantime, genetic variants have been identified in further downstream genes such as *PMEL*, which, for example, changes the basic coat colour of Charolais cattle to the breed‐specific light or dilute form (OMIA 001545‐9913; Kühn & Weikard, 2007). In a group of Holstein cattle with red coat colour but without *MC1R* variation, heterogeneity at or interaction with other loci was indicated to be responsible for this dominant red coat colour (Lawlor et al., 2014). Later, the exact cause for this *COPA*‐related dominant inherited variant red phenotype (OMIA 001529‐9913) became known (Bourneuf et al., 2017; Dorshorst et al., 2015). Finally, in addition to the three bovine *E* alleles mentioned above, two further protein‐changing *MC1R* variants, denoted *E^d1^* and *E^d2^*, have been identified in cattle, but neither for the p.Arg223Trp nor for the p.Gly220_Arg223dup variant can a clear influence on the base colour be demonstrated so far (Graphodatskaya et al., 2002; Kriegesmann et al., 2001; Rouzaud et al., 2000). In domestic yaks, two *MC1R* missense variants, p.Gln114Lys and p.Ala291Thr, which occur in perfect linkage disequilibrium, have been associated with dominantly inherited black, so‐called imperial, nose pigmentation (OMIA 001199‐30521; Petersen et al., 2020). In Kumamoto cattle, a recessive form of *MC1R*‐related solid light brown observed in Japanese Brown cattle is caused by a p.Ala291Thr missense variant affecting the seventh transmembrane domain of MC1R (Matsumoto et al., 2020). Recently, the p.Thr281Asn variant in this highly conserved protein region was reported to cause a recessive form of *MC1R*‐associated red coat colour in Sahiwal, an indicine breed of cattle (OMIA 001199‐9915; Goud et al., 2021). In this study, the genetic basis for black as well as red pheomelanin phenotypes was evaluated using a genome‐wide association in Evolèner, Eringer and Valdostana cattle, three related Alpine cattle breeds of Switzerland and Italy.

Eringer (Hérens in French) is an old cattle breed from the canton of Valais in Switzerland and its current population includes fewer than 17 000 female herd book individuals (Signer‐Hasler et al., 2017). The most popular colour is the solid black coat, but animals of any colour, including red and dark brown or black, occur (Lauvergne et al., 2004). Eringer are characterised as small and strong cattle with a brachycephalic head (Felius, 1995). The name of the Evolèner (Evolénarde in French) breed originates from the village Evolène in the municipality Val d’Hérens in the canton Valais. Historically, the breed was constituted from Eringer cattle showing a pied coat pattern, characterised by white markings particularly on their foreheads, while their basic coat colour ranges from red to black. Recently we reported that the *KIT^PINZ^* variant occurs in some white‐spotted animals of Evolèner and Eringer, confirming admixture and the reported historical relationship of these breeds (Küttel et al., 2019). Today’s Evolèner population represents the most endangered autochthonous cattle breed, with fewer than 500 female herd book individuals (Signer‐Hasler et al., 2017; https://www.blw.admin.ch/blw/en/home/nachhaltige‐produktion/tierische‐produktion/tierzucht‐und‐tiergenetische‐ressourcen.html). Since animals from the geographically neighbouring Italian Aosta valley are also sporadically kept in Valais, we also included Valdostana cattle in this study. Italian Valdostana are managed in two herd books (Mastrangelo et al., 2020; Mazza et al., 2015; Strillacci et al., 2020). The coat colour is black or wild‐coloured (sometimes called chestnut) in Pezzata Nera and Castana (VPN–CAST) and red in Pezzata Rossa (VPR). Recent studies confirmed a closer genetic relationship between the CAST and VPN subpopulations, probably attributable to repeated crossbreeding between Eringer cattle from Switzerland and the VPN that have generated the CAST (Forabosco & Mantovani, 2011; Mastrangelo et al., 2020).

A total of 513 cattle, comprising 337 Eringer, 144 Evolèner and 32 Valdostana, were considered for this study (Table S1). Based on photographs, the animals were divided into three colour groups: 216 black, 76 red and 221 wild‐coloured individuals (Figure S1c). Only cattle that are completely black, including the udder and teat skin, belong to the black phenotype. Red animals are solid red with different shades and/or intensities, but without visible black areas. The wild‐coloured group is more variable, as the cattle express both pigments, which means that both colours are present, but not always in the same intensity and to the same extent. Furthermore, cattle in the latter group all exhibit less intense pigmentation of the skin of the udder or scrotum (Figure S1c).

A subset of 396 cattle, including animals of all three breeds and base coat colour (Table S1; Figure S1b), were genotyped either with the Illumina BovineHD BeadChip array for 777k markers or with the GeneSeek Genomic Profiler Bovine Array for 134k markers. Date merging and filtering were carried out using plink software version 1.9 (Chang et al., 2015). Only markers occurring in both arrays and with known positions in the ASR‐UCD1.2 reference genome assembly (Rosen et al., 2020) were retained. Quality control included filtering for locus genotyping rate (>90%). Finally, 114 643 autosomal SNPs were included in the genome‐wide association study. The study was performed using a linear mixed model while adjusting for population stratification as implemented in gemma software version 0.98.1 (Zhou & Stephens, 2012). The genome‐wide significance threshold was estimated by Bonferroni correction of a nominal *p* threshold of 0.05 for 114 643 simultaneous tests, yielding a −log_10_ (*p*) threshold of 6.36 × 10^−7^. The results were plotted using r (https://www.R‐project.org/). A strong single genome‐wide significant association signal located on chromosome 18 was obtained (Figure S1a). The five markers all had a value of less than 8 × 10^−31^ and were located between 14 208 633 and 14 988 936 bp flanking the *MC1R* gene, which maps to 14.7 Mb (Table S2).

To gain insight into the molecular basis on coat colour variation within these Alpine breeds, we amplified the entire coding sequence of the bovine *MC1R* gene with two overlapping genomic PCR products: for the 5′‐region, a 772 bp‐sized amplicon using a forward primer starting at 14 705 197 bp on chromosome 18 (F1, 5′‐AGGCGGACTGAGAACAGAAG‐3′) in combination with a reverse primer starting at 14 705 968 bp (R1, 5′‐GCTATGAAGAGGCCAACGAG‐3′); and for the 3′‐region, a 903 bp‐sized amplicon using a forward primer starting at 14 705 793 bp on chromosome 18 (F2, 5′‐GTGGACCGCTACATCTCCAT‐3′) in combination with a reverse primer starting at 14 706 694 bp (R2, 5′‐CCAGTCACCACAGAGCGTTA‐3′). PCR products were directly sequenced on an ABI 3730 capillary sequencer (Thermofisher) after treatment with exonuclease I and shrimp alkaline phosphatase. Sequence data were analysed using sequencher 5.1 (GeneCodes).

Sequencing of the entire coding sequence of *MC1R* in all 513 cattle revealed two additional protein‐changing variants in addition to the five previously reported functional *MC1R* alleles *E* ^+^, *E^D^*, *e*, *E^d1^*, and *E^d2^* (Table 1; Figure 1). The first newly detected variant, denoted as *e^v1^*, can be designated as Chr 18: 14 705 799C>T (ASR‐UCD1.2). It is a missense variant, NM_174108: c.424C>T, predicted to change a highly conserved arginine residue in the third transmembrane domain of the melanocortin‐1 receptor, NP_776533: p.Arg142Cys (Figure 1d). The second variant, denoted as *e^v2^*, can be designated as Chr 18: 14 705 638G>A (ASR‐UCD1.2). It is a missense variant, NM_174108: c.263G>A, predicted to change a highly conserved serine residue in the second transmembrane domain of MC1R, NP_776533: p.Ser88Asn (Figure 1d).

**TABLE 1:** Summary of the *MC1R* variants and their *E* allele designation, and the observed haplotype diversity in 513 individuals from three Alpine cattle breeds

Allele		*e^v2^*	*E^D^*	*e*	*e^v1^*	*E^d1^*	*E^d2^*
ARS‐UCD1.2		14705638G>A	14705671T>C	14705686delG	14705799C>T	14706042C>T	14706033_14706045dupGGCATTGCCCGG
NM_174108		c.263G>A	c.296T>C	c.310delG	c.424C>T	c.667C>T	c.658_669dupGGCATTGCCCGG
NP_776533		p.Ser88Asn	p.Leu99Pro	p.Gly104ValfsTer53	p.Arg142Cys	p.Arg223Trp	p.Gly220_Arg223dup
Haplotype	Frequency						
*E^+^*	0.371	G	T	G	C	C	wt
*E^+d1^*	0.041	G	T	G	C	T	wt
*E^+d2^*	0.088	G	T	G	C	C	dup
*E^D^*	0.244	G	C	G	C	C	wt
*E^Dd2^*	0.016	G	C	G	C	C	dup
*e*	0.101	G	T	del	C	C	wt
*e^d2^*	0.008	G	T	del	C	C	dup
*e^v1^*	0.089	G	T	G	T	C	wt
*e^v1d1^*	0.009	G	T	G	T	T	wt
*e^v1d2^*	0.004	G	T	G	T	C	dup
*e^v2^*	0.025	A	C	G	C	C	wt
*e^v2d1^*	0.004	A	C	G	C	T	wt

Both predicted non‐conservative exchanges affect conserved residues in MC1R orthologues across phylogenetically diverse vertebrate species (Figure 1c). The *MC1R* missense variants were predicted to be deleterious using provean software (Choi & Chan, 2015). Missense variants in transmembrane domains were previously reported to impair the function of the melanocortin 1 receptor (Goud et al., 2021; Matsumoto et al., 2020). Like the causal missense variants found in the red Kumamoto and the red Sahiwal cattle respectively, the two missense variants described herein both also affect conserved residues, but in a different transmembrane domain of MC1R (Figure 1d). We assume that the change from the hydrophilic arginine to the hydrophobic cysteine at position 142 of the as *e^v1^* allele is intolerable for the integration of the protein in the membrane, thus causing a complete loss‐of‐function of MC1R. Furthermore, the introduction of a cysteine residue may give rise to the formation of disulphide bonds and thus change the secondary and tertiary structure of the receptor molecule. In the *e^v2^* allele, the exchange of serine for asparagine at position 88 within the second transmembrane domain replaces a small polar side chain for another that is chemically similar but physically larger and has the potential to form additional hydrogen bonds. Genotyping revealed that the *e^v2^* allele occurs in perfect linkage disequilibrium with *E^D^* (Table S1; Table 1). Although only two homozygous red‐coloured animals could be found for this rare allele, all 22 compound heterozygous *e^v2^*/*e* or *e^v1^*/*e^v2^* cattle were red (Table 2). Therefore, we suspect that this variant overcompensates for the gain of function of the p.Leu99Pro variant and represents a partial loss‐of‐function mutation that arose later on top of the derived *E^D^* allele. One can speculate that the receptor no longer responds to the ASIP protein, although a residual function might remain, which could explain the slightly more intense chestnut‐like red coat colour compared with the animals homozygous for the loss‐of‐function allele *e*. Interestingly the carriers of at least one *e^v1^* allele seem to have darker coat and muzzle than animals carrying the other two recessive alleles *e* or *e^v2^* (Figure 1a).

**TABLE 2:** Association of *MC1R* diplotypes with base coat colour in 513 Alpine cattle of three breeds

*MC1R*	*n*	Coat colour			Breed		
Diplotype		Wild‐coloured	Black	Red	Eringer	Evolèner	Valdostana (VPN/CAST)
*E^D^/E^+^*	112	2	110		104	3	5
*E^+^/E^+^*	74	74			67	5	2
*E^D^/E^D^*	46		46		32	3	11
*E^+^/E^+d2^*	45	45			38	4	3
*E^+^/e*	29	29			19	7	3
*e^v1^/e^v1^*	20			20		20	
*e^v1^/e*	18			18		18	
*E^D^/e*	16		16		8	5	3
*e^v2^/e^v1^*	13			13		13	
*E^D^/E^+d2^*	13	1	12		11	2	
*E^Dd2^/E^+^*	13	1	12		11	2	
*E^+d1^/E^+^*	13	13			8	3	2
*E^+^/e^v1^*	11	11			1	10	
*E^D^/E^+d1^*	9		9		8		1
*e/e*	9			9	2	6	1
*e^v2^/e*	9			9		9	
*E^+d1^/e*	8	8			3	5	
*E^+d2^/E^+d2^*	8	8			8		
*E^D^/e^v1^*	6		6			6	
*E^+^/e^d2^*	5	5			3	1	1
*E^+d1^/e^v1d1^*	5	5				5	
*E^+d1^/E^+d2^*	5	5			4	1	
*E^+d2^/e*	4	4			3	1	
*E^+^/e^v2d1^*	4	4				4	
*E^Dd2^/E^+d2^*	3		3		2	1	
*E^+d2^/e^d2^*	3	3			3		
*e^v1d2^/e^v1^*	2			2		2	
*E^D^/e^v2^*	2		2		1	1	
*e^v1d2^/e*	1			1		1	
*e^v1^/e^d2^*	1			1		1	
*E^+d2^/e^v1d2^*	1	1				1	
*e^v2^/e^v2^*	1			1		1	
*e^v2d1^/e^v2d1^*	1			1		1	
*E^+d1^/E^+d1^*	1	1			1		
*e^v1d1^/e*	1			1		1	
*E^+^/e^v1d1^*	1	1				1	
Total	513	221	216	76	337	144	32

Manual phasing of the genotypes of all 513 cattle for these seven *E* alleles revealed evidence of 12 different *MC1R* haplotypes occurring at different frequencies (Table 1). The two mostly likely neutral alleles, *E^d1^* and *E^d2^*, not only occurred in association with the ancestral *E* ^+^ allele, denoted as *E^+d1^* and *E^+d2^*, but rarely also occurred in linkage with four other *E* alleles. Thus, *E^d2^* was found to be associated with *E^D^*, *e* and *e^v1^*, denoted as *E^Dd2^*, *e^d2^* and *e^v1d2^*, as well as *E^d1^* in combination with *e^v1^* and *e^v2^*, denoted as *e^v1d1^* and *e^v2d1^* respectively. The diverse combination of these 12 haplotypes resulted in a total of 36 different diplotypes in the 513 genotyped cattle (Table 2). Whether this unexpected heterogeneity can be explained by recombination or recurrent mutations is not yet clear. Nevertheless, we found a perfect association of the genotypes with the coat colour phenotypes (Table 2). Without exception, all 216 black animals carried at least one copy of the known dominant *E^D^* allele, while the 76 red animals were either homozygous or compound heterozygous for one of the three recessive alleles *e*, *e^v1^* and *e^v2^* (Table 2; Figure S1c). Interestingly, the largest group of cattle had a wild coat colour and was genetically the most diverse group with 19 different diplotypes. In addition to 74 animals homozygous for *E* ^+^/*E* ^+^, we observed 10 different diplotypes including heterozygotes for all different versions of the three red‐associated recessive alleles (Table 2; Figure S1c). Four out of 221 wild‐coloured cattle were heterozygous *E^D^*/*E* ^+^, which represents a discordance between genotype and phenotype (Table 2; Figure S1c). We revisited these animals and confirmed that they were obviously not black. Additional genetic factors such as additional variants in the regulatory region of *MC1R* could interfere with the effect of the gain‐of‐function allele, just as the *e^v2^* allele interferes with the dominant effect of *E^D^*. Rouzaud et al. (2000) speculated that the *E^d2^* that they denoted as *E^1^* could be responsible for the attenuated coat colour of Aubrac and Gasconne cattle. In line with previous reports, we could not find evidence for an impact on coat colour of the protein‐changing variants associated with *E^d1^* and *E^d2^* (Table 2). Maybe these variants are functionally neutral because they are located in an intracellular loop between two transmembrane domains and therefore do not affect the function of the receptor.

In the three Alpine cattle breeds initially considered, the two newly discovered alleles occurred primarily in the Evolèner population (Table 3). The allele frequency in the study cohort, comprising 144 Evolèner cattle, was 0.35 for *e^v1^* and 0.11 for *e^v2^*. Both alleles occurred only sporadically in more than 300 Eringer individuals, while they were absent in the 32 VPN–CAST cattle. However, owing to the breeding history, we expect to observe carriers for these two new alleles in this breed when a larger number of samples, including the red VPR, can be examined. As both variants were added to custom genotyping arrays used in routine SNP genotyping for genomic selection in Switzerland and France, we were able to observe the occurrence of these two alleles in expanded cohorts of other cattle breeds as well as other cohorts of 54 Eringer and 202 Evolèner, resulting in almost identical allele frequencies (Table 3). In addition, we searched for the presence of both alleles in the recent variant catalogues of the ReDiverse (Biodiversity within and between European Red Dairy Breeds; https://era‐susan.eu/content/rediverse) and the 1000 Bull Genomes projects (Hayes & Daetwyler, 2019), revealing single heterozygous carriers for both variants in other cattle of various breeds (Table 3). Besides Evolèner, the *e^v1^* allele was found in other cattle breeds with red coat colour, such as the Local breed of Belgium, Gloucester, German Red Angler and Salers. The *e^v2^* allele occurred in Eastern Finncattle, Kalmyk, Tarentaise, Simmental and Abondance (Table 3). Genotyping of a cohort of 2186 French Abondance revealed an allele frequency of 0.07 for *e^v2^* and in the German Rotes Höhenvieh cattle this allele segregates at an allele frequency of 0.09 (Table 3). Both breeds have a characteristic solid intense red (chestnut‐like) coat colour, apparently determined by two different *MC1R* loss‐of‐function alleles, *e* and *e^v2^*. In a large cohort of Simmental, Brown Swiss and Holstein cattle from Switzerland and France, the *e^v2^* occurred only very rarely, with frequencies below 0.005, whereas the *e^v1^* was noticed in single animals of Angus, Original Braunvieh, Brown Swiss, Simmental, Holstein, Normande and Limousin breeds (Table 3). Besides the obvious clustering in the Evolèner breed, both of the newly discovered variants occur with varying frequencies in numerous geographically close populations such as Abondance, Tarentaise and Simmental, as well as several more distant breeds. No carriers for the two newly discovered alleles were found in the examined selection of Austrian Pinzgauer and Tux‐Zillertaler cattle, as well as the German dual‐purpose Rotbuntes Niederungsrind and the related Dutch Meuse–Rhine–Yssel (Table 3). The occurrence and frequencies of variants in the *MC1R* gene may be an indication of phylogenetic relationships between different European breeds, as previously noted (Kasprzak‐Filipek et al., 2020). Presumably, these mutation events occurred before the formation of today’s breeds. Whether the high allele frequency in the Evolèner points to the origin of these two additional coat colour‐associated recessive *MC1R* variants remains unclear. Nevertheless, the *e^v2^* mutation might have originated in the Alpine arch, as it is now more common in the Evolèner than in the neighbouring Abondance and Rotes Höhenvieh cattle.

**TABLE 3:** Occurrence of the *MC1R* alleles *e^v1^* and *e^v2^* in different cattle breeds

Breed	Source of data	*e^v1^* (c.424C>T)			*e^v2^* (c.263G>A)		
		*CC*	*CT*	*TT*	*GG*	*GA*	*AA*
Eringer	This study	336	1		336	1	
Evolèner	This study	65	57	22	115	27	2
Valdostana	This study	32			32		
Holstein	Switzerland	19 295	40		13 577	26	
Brown Swiss	Switzerland	18 746	45		12 034	49	
Original Braunvieh	Switzerland	3567	7		2056	4	
Simmental	Switzerland	2555	2		1942	17	
Limousin	Switzerland	1202	1		1140		
Angus	Switzerland	678	1		632		
Evolèner	Switzerland	88	93	21	171	31	1
Eringer	Switzerland	53	1		54		
Pinzgauer	Switzerland/Austria	140			140		
Tux‐Zillertaler	Switzerland/Austria	49			49		
Holstein	France	96 660	2		96 661	1	
Normande	France	12 976	1		12 977		
Abondance	France	2186			1871	309	6
Simmental	France	2120			2095	25	
Tarentaise	France	1226			1226		
Salers	France	384			384		
Rotes Höhenvieh	Germany/Denmark^a^	101			84	17	
Rotbuntes Niederungsrind (MRY)	Germany/The Netherlands^a^	38			38		
Eastern Finncattle	Finland/Denmark^a^	12			10	2	
Eastern Flanders White‐Red	Belgium/Denmark^a^	9	1		10		
Red Holstein	Germany^a^	107	1		108		
>120 global breeds	1000 Bull Genomes project (run 9)	5043	8^b^		5035	9^c^	

^a^ https://era‐susan.eu/content/rediverse.

^b^ Evolèner (*n* = 2), unknown Chinese (*n* = 2), Eastern Flanders White Red (*n* = 1), Gloucester (*n* = 1), German Red Angler (*n* = 1), Salers (*n* = 1).

^c^ Eastern Finncattle (*n* = 3), Kalmyk (*n* = 2), Tarentaise (*n* = 1), Simmental (*n* = 1), Abondance (*n* = 1), unknown US (*n* = 1).

In conclusion, the discovery of further recessive alleles for red coat colour in cattle expands the spectrum of causal *MC1R* loss‐of‐function variants. The detected heterogeneity supports the assumption that the numerous derived alleles are most likely very old. We propose the following order of dominance for the five functional bovine *MC1R* alleles: *E^D^* > *E* ^+^ > *e* = *e^v1^* = *e^v2^*. As reported in other species, non‐coding regulatory changes in other genes, including, e.g., *ASIP*, *KITLG* and *PMEL*, could additionally influence the intensity of the red colour.

## CONFLICT OF INTEREST

The authors declare that they have no competing interests.

## Supporting information

Fig S1

Table S1

Table S2

## Data Availability

The data supporting the findings of this study can be found in the electronic appendix (Supporting Information) for this publication. SNP genotyping data can be retrieved at https://osf.io/pjm69/.
