# Supplementary material for: Identification of two new recessive MC1R alleles in red‐coloured Evolèner cattle and other breeds
Source: Anim Genet. 2022 Apr 22;53(3):427–35. doi: 10.1111/age.13206 (PMC9373916; doi:10.1111/age.13206)
Supplement: Supplementary file 1 — Fig S1 [file AGE-53-427-s001.docx]

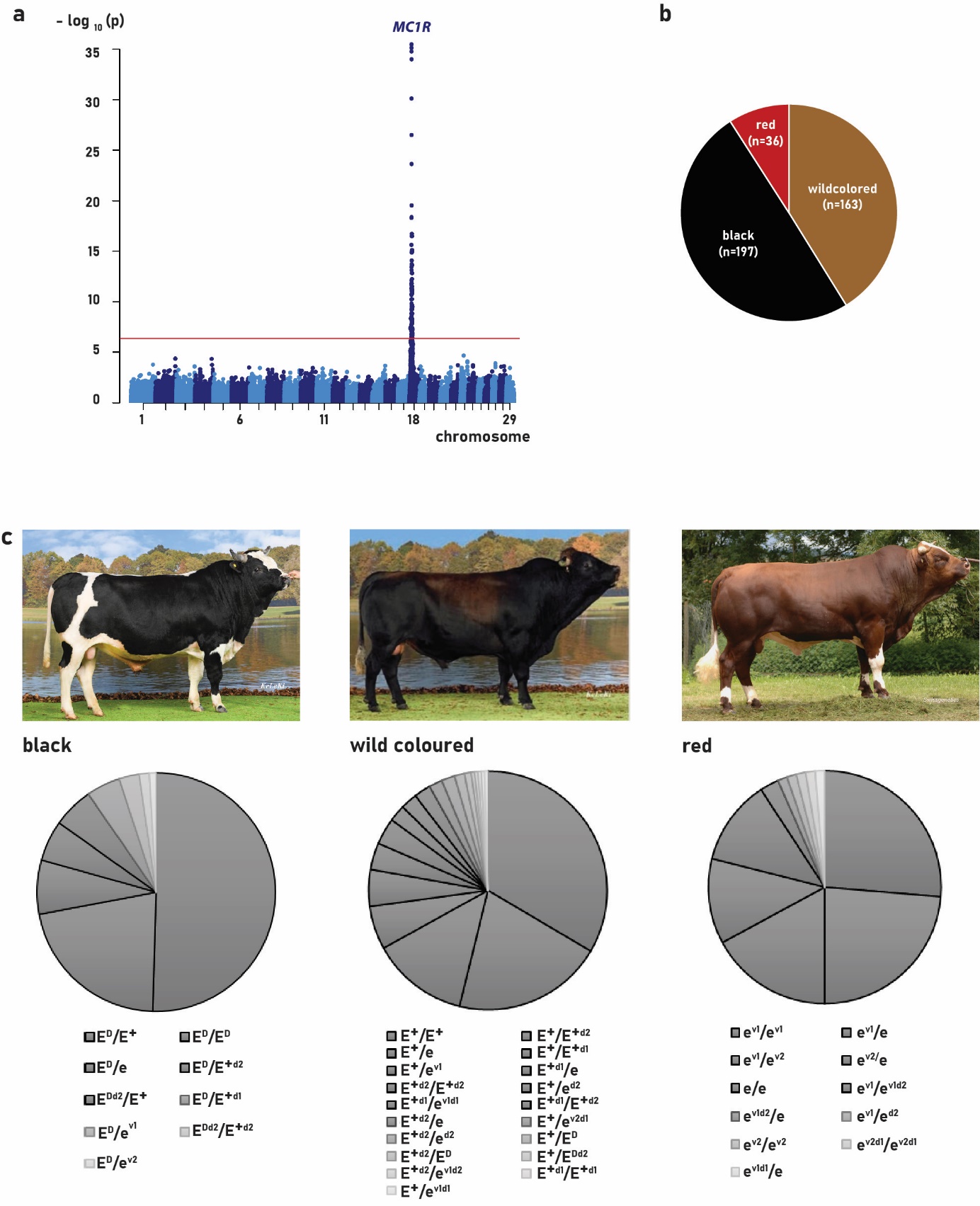
**Figure S1** The base colour of the Alpine cattle breeds studied can be explained by a pronounced diversity at the *MC1R* locus. (a) Manhattan plot of -log(*P*-values) for the genome wide association study shows a genome wide significant associated locus on cattle chromosome 18 in the region of the *MC1R* gene. The red line represents the Bonferroni-corrected significance threshold (−log(*P*-value) = 6.36). (b) Distribution of coat colour among the 396 cattle from the GWAS. (c) Representative photos illustrating the three different coat colour types. Pie charts illustrating the heterogeneity within the three colour groups studied. The different *MC1R* diplotypes are shown in different shades of grey (9 different diplotypes were found for black coloured animals, 19 different diplotypes for wild coloured and 11 different diplotypes for red animals).
